# A comparison of 71 binary similarity coefficients: The effect of base rates

**DOI:** 10.1371/journal.pone.0247751

**Published:** 2021-04-07

**Authors:** Michael Brusco, J. Dennis Cradit, Douglas Steinley

**Affiliations:** 1 Department of Business Analytics, Information Systems, and Supply Chain, Florida State University, Tallahassee, Florida, United States of America; 2 Department of Psychological Sciences, University of Missouri, Columbia, Missouri, United States of America; Shandong University of Science and Technology, CHINA

## Abstract

There are many psychological applications that require collapsing the information in a two-mode (e.g., respondents-by-attributes) binary matrix into a one-mode (e.g., attributes-by-attributes) similarity matrix. This process requires the selection of a measure of similarity between binary attributes. A vast number of binary similarity coefficients have been proposed in fields such as biology, geology, and ecology. Although previous studies have reported cluster analyses of binary similarity coefficients, there has been little exploration of how cluster memberships are affected by the base rates (percentage of ones) for the binary attributes. We conducted a simulation experiment that compared two-cluster *K*-median partitions of 71 binary similarity coefficients based on their pairwise correlations obtained under 15 different base-rate configurations. The results reveal that some subsets of coefficients consistently group together regardless of the base rates. However, there are other subsets of coefficients that group together for some base rates, but not for others.

## Introduction

Two-way, two-mode data are extremely common in psychology and other areas of scientific inquiry. The two-way nature of data pertains to their arrangement in a two-dimensional array, where there are measurements for each row and column of the array. The two-mode aspect of the data relates to the fact that the *n* rows and *p* columns of the *n* × *p* two-dimensional array correspond to two distinct sets of objects. In psychological contexts, it is particularly common for the row objects to be *individuals* (e.g., patients, examinees, respondents, etc.), and the column objects to be *attributes* (e.g., symptoms, test questions, survey items, etc.).

Our focus in this paper is on two-mode *binary* data. The data are arranged in a two-dimensional array, **X** = [*x*_*ij*_], where *x*_*ij*_ = 1 if attribute *j* is affirmatively measured for individual *i* and *x*_*ij*_ = 0 if attribute *j* is not affirmatively measured for individual *i*, for all 1 ≤ *i* ≤ *n* and 1 ≤ *j* ≤ *p*. The psychological literature is replete with examples of two-mode binary data. For example, in an educational testing context, *x*_*ij*_ = 1 could correspond to examinee *i* providing a correct response to test question *j*. Likewise, in a psychopathology setting, *x*_*ij*_ = 1 might reflect the presence of symptom *j* for patient *i*.

In this paper, our focus is on the analysis of the attributes, which is especially relevant to psychological applications such as item-scale development in exploratory Mokken scaling analysis [1–4] and network analysis of symptoms in psychopathology [5]. In these and other applications, it is common for the number of individuals to far exceed the number of attributes (i.e., *n* >>>*p*). Therefore, when the focus is on the attributes, a typical starting point is to establish *binary similarity coefficients* that measure inter-attribute similarity. To maintain greater clarity, we limit our focus in this paper to binary *similarity* coefficients where larger coefficient values reflect greater similarity. Although less common, there are also binary *dissimilarity* coefficients, whereby larger coefficient values indicate less similarity. In most instances, coefficients can be transformed from similarity to dissimilarity (or vice versa) by taking one minus the coefficient value. The problem of specifying binary similarity coefficients has been studied for more than 100 years, spanning the pioneering development of the earliest coefficients [6–9], comparative studies in the 1980’s [10–12], and several surveys in the last dozen years [13–16].

Table 1 displays the standard convention for the presentation of binary similarity coefficients. The four cells of the table correspond to all possible pairings of binary measurements for two attributes *j* and *l*. The value of *a* is a count of the number of matches of 1s for *j* and *l* (i.e., *x*_*ij*_ = *x*_*il*_ = 1) across all *n* respondents. Likewise, the value of *d* is a count of the number of matches of 0s for *j* and *l* (i.e., *x*_*ij*_ = *x*_*il*_ = 0) across all *n* respondents. Some authors refer to matches of 1s as *presence* matches and matches of 0s as *absence* matches [11]. Other authors use the terms *positive* and *negative* matches to refer to matches of 1s and 0s, respectively [13]. The values of *b* and *c* are counts of mismatches between *j* and *l* across all *n* respondents. Measure *b* is a count (across all 1 ≤ *i* ≤ *n*) of mismatches where *x*_*ij*_ = 1 and *x*_*il*_ = 0, and measure *c* is a count (across all 1 ≤ *i* ≤ *n*) of mismatches where *x*_*ij*_ = 0 and *x*_*il*_ = 1.

**Table 1:** Contingency table structure for two binary attributes (*j* and *l*) measured across 1 ≤ *i* ≤ *n* observations.

	Attribute *l* assuming a value of 1	Attribute *l* assuming a value of 0
Attribute *j* assuming a value of 1	*a* = number of positive matches *a* = $\sum_{i=1}^{n}x_{ij}x_{il}$	*b* = number of mismatches (attribute *j* occurrence) *b* = $\sum_{i=1}^{n}x_{ij}(1-x_{il})$
Attribute *j* assuming a value of 0	*c* = number of mismatches (attribute *l* occurrence) *c* = $\sum_{i=1}^{n}(1-x_{ij})x_{il}$	*d* = number of negative matches *d* = $\sum_{i=1}^{n}\left(1-x_{ij})(1-x_{il}\right)$

It is abundantly clear from the literature that some binary similarity coefficients are quite familiar to psychological researchers, whereas many others are virtually unknown. However, it is important to note that these same coefficients, despite relative unfamiliarity among psychological researchers, are actively used and well known in areas such as chemistry [14], ecology [12], and bioinformatics [16]. In light of the vast array of binary similarity coefficients, it is helpful to ascertain how coefficients tend to group together. In [13], the authors randomly generated binary vectors and computed 76 coefficients for each pair of vectors. An agglomerative hierarchical cluster analysis (using single linkage) of the coefficients was performed based on their pairwise correlations across 100 trials. The precise details of the generation of the data sets was not provided. Later, in [14], researchers conducted a multidimensional scaling analysis of binary similarity coefficients based on their pairwise correlations obtained across 100,000 trials. Rather than generate binary vectors, these authors randomly generated values for *a*, *b*, *c*, and *d*, while assuring that they summed to a fixed constant. This data generation process resulted in rather extreme conditions across the 100,000 trials.

**Fig 1:**
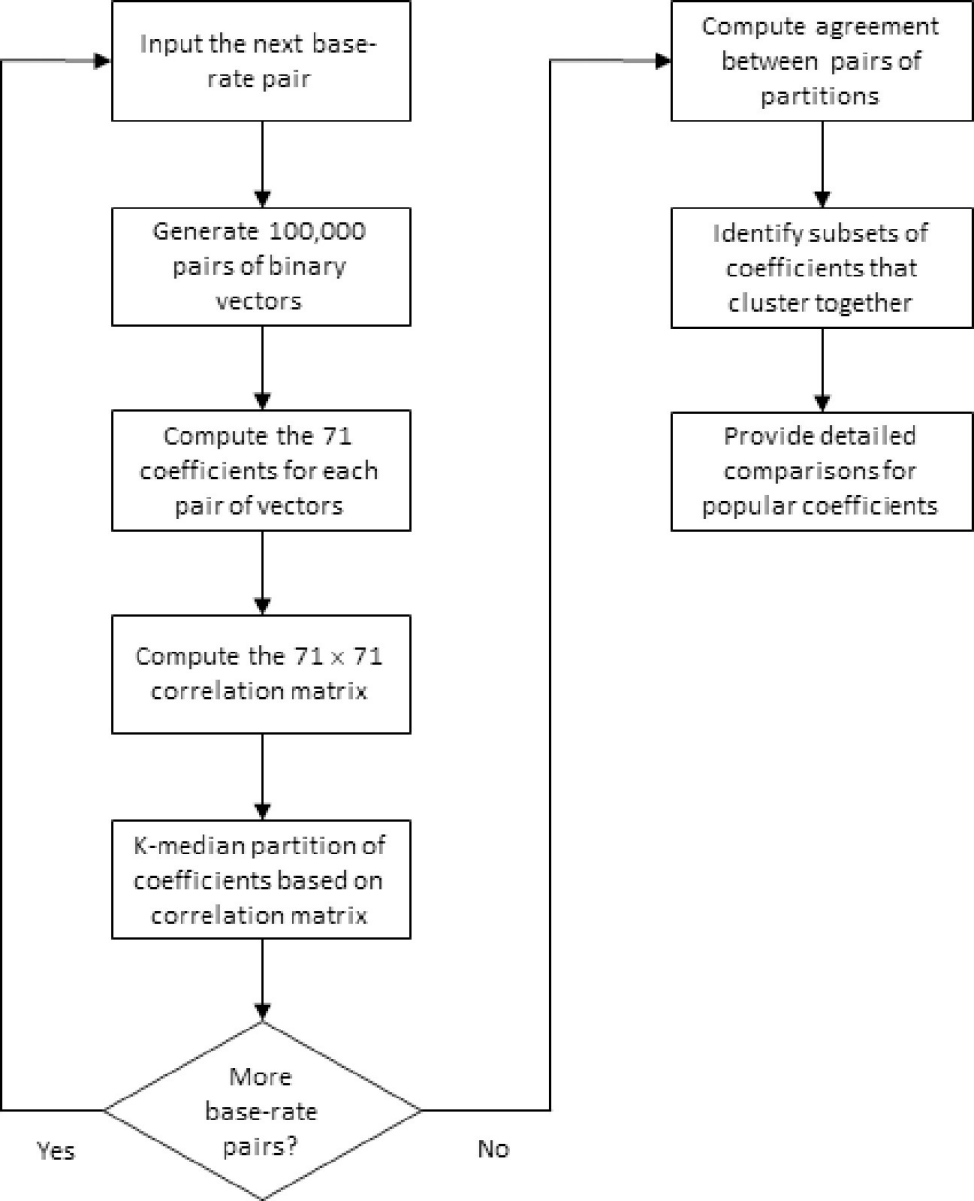
A summary of the workflow of the experimental study.

Neither of these previous studies provided any insight as to how the relative agreement of binary similarity coefficients is affected by the *base rates* of the binary vectors. The base rate for a binary vector is simply the percentage of ones in the vector. We hypothesize that, although the concordance between some binary similarity coefficients might be unaffected by the base rates, the agreement between other coefficients could be profoundly affected. Accordingly, we conducted a simulation analysis that systematically evaluated the effect of base rates on the grouping of 71 binary similarity coefficients. A summary of the workflow associated with this simulation analysis is provided in Fig 1. This required an experimental design that explicitly controlled for the base rates by establishing a design level corresponding to a pair of base rates for the binary vectors. A separate partitioning analysis was completed for each design level.

The partitioning method that we have selected for our analysis is *K*-median partitioning [17–20]. The rationale for selecting this method was based, in large part, on the fact that *K*-median partitioning is flexible and can accommodate either similarity or dissimilarity proximity data with relative ease [21–23]. By contrast, the popular *K*-means partitioning method [24,25] is designed for dissimilarity data based on squared Euclidean distances.

In the next section, we discuss the selection of the 71 binary similarity coefficients that are considered in our comparative analyses. This is followed by a section that describes the *K*-median partitioning problem and the heuristic that we use to obtain solutions. We then use the *K*-median heuristic to produce a partition of the 71 binary similarity coefficients based on their inter-coefficient correlations established from a large number of simulated trials. This is followed by a conclusion section that summarizes the paper, provides recommendations, and discusses the limitations and extensions.

## Binary similarity coefficients

There are several different possible schemes that can be used to categorize binary similarity coefficients. For example, one classification scheme [12,26] divides coefficients into two categories: co-occurrence and association. Co-occurrence coefficients typically range from 0 to 1, and often have *a* (or *a*+*d*) in their numerator. Thus, they are largely determined by the frequency of occurrences for the attributes. By contrast, association coefficients typically range from -1 to +1, and often have (*ad*–*bc*) in their numerator. In [12], it was posited that association coefficients have an inherent centering effect that makes them less vulnerable to *size effects* that can occur with co-occurrence coefficients. A size effect in our context would pertain to the resulting partitions being overly sensitive to the relative frequencies of 1s in the data.

A second classification scheme for binary similarity coefficients pertains to the inclusion, exclusion, or differential weighting of negative matches (*d*) in the computation of the coefficient. Many coefficients (including all of the association coefficients) include *a*, *b*, *c*, and *d* in their computation. However, there are also coefficients that either exclude *d* entirely in the computation [6,27,28], or include *d* but reduce its contribution to the index relative to *a* [29]. These are sometimes referred to as *asymmetric* coefficients. The motivation for excluding (or diminishing the contribution of) *d* has its foundation in the principle that *shared presences* are more informative than *shared absences* in ecological/biological data [26,29,30]. In fact, it had been suggested that negative matches might not reflect any similarity between attributes at all [13,31]. On the other hand, in [10] it was have noted that some applications do not use 1 and 0 to indicate, respectively, the presence or absence of an attribute, but rather qualitative differences of equal status (e.g., male or female, married or single, working or retired).

Based primarily on two of the most recent surveys [13,14], we assembled 71 binary similarity coefficients for evaluation in our analyses. An effort was made in the selection process to exclude coefficients that were obviously identical to other coefficients. Typically, this occurs because some coefficients are called by different names. For example, the popular Jaccard coefficient is sometimes referred to as the Tanimoto coefficient. Likewise, the closely-related Gleason coefficient is also known as the Czekanowski, Dice, Sørensen, and Sørensen-Dice coefficient. The 71 coefficients are provided in the Appendix.

## Model and method for *K*-median partitioning

### Model formulation

A partition of the attributes into *K* clusters can be established for each similarity matrix using *K*-median partitioning [17,18], which is also sometimes known as *p*-median partitioning [20] or partitioning around medoids [19]. Denoting the *p* attributes via the index set *P* = {1,…, *p*}, *K*-median partitioning seeks to identify a subset *Q* that consists of *K* representative attributes (known as exemplars), and to assign each attribute to its most similar exemplar, such that the sum (across all attributes) of the similarities between each attribute and its nearest exemplar is maximized. A succinct mathematical statement of the *K*-median problem in dissimilarity form was provided in [32], which was adapted to the similarity context in [23]. For a given similarity matrix **S** = [*s*_*ij*_], the mathematical formulation is as follows:

$$\underset{Q}{Maximize}:\;Z=\sum_{i\in P}\left[\underset{j\in Q}{\max}\{s_{ij}\}\right],$$ (1)

subjectto:Q⊂P, (2)

|Q|=K. (3)

Eqs (2) and (3) are constraints that assure, respectively, that *Q* is a subset of the set of attribute indices and the number of indices in *Q* (denoted by |*Q*|) is equal to the desired number of clusters, *K*. Eq (1) is the objective function, which seeks the particular subset *Q* that maximizes the sum (across all attributes) of the similarity between the attribute and the exemplar to which it is most similar. It is assumed that *s*_*jj*_ is the largest element in row *j* (for all 1 ≤ *j* ≤ *p*), which assures that, if attribute *j* is selected as an exemplar, then attribute *j* will be assigned to the cluster for which it is the exemplar.

### Solution methods and computer implementation

An optimal solution to the optimization problem posed by Eqs (1–3) can be obtained by re-formulation and solution via integer linear programming [20,33–35]. Methods based on Lagrangian relaxation and branch-and-bound programming have also proven to be computationally effective [22]. Numerous heuristic procedures have also been developed (see [32] for a review). An effective and efficient fast interchange heuristic method was proposed in [36] and later improved in [37,38]. A multistart implementation of this procedure has been used for application to real datasets in [21,23], as well as for a recent large-scale simulation study [39]. We used this multistart heuristic for obtaining attribute partitions in the subsequent sections of this paper. The steps of the procedure are as follows:

1. Randomly choose *K* attributes as initial exemplars.
2. Place each attribute in the cluster associated with its nearest exemplar.
3. Evaluate the replacement of each exemplar with one of the attributes not currently selected as an exemplar. If a replacement increases the sum of the similarities between the attributes and their most similar exemplar (i.e., *Z* in Eq (1)), then that replacement should be accepted.
4. Repeat Step 3 until no exemplar replacement will further increase *Z*.

The fast interchange heuristic does not guarantee a globally-optimal solution; however, the solution is locally-optimal in terms of all possible replacements of an exemplar with an attribute not chosen as an exemplar. A recommendation of restarting the algorithm 2000 times and adopting the best solution across the 2000 restarts was proposed in [21] and was used in our analyses. Several studies support the notion that this multistart fast interchange procedure will provide good (and frequently globally-optimal solutions) for the size of *K*-median problems commonly encountered in psychology [21,23,38]. For problems where *K* exceeds 10, we recommend the use of metaheuristics for *K*-median clustering (see [32] for a review).

## Simulation experiment

### Data generation process

Our primary interest in this paper is on the effect that base rates have on partitions of binary similarity coefficients. This requires an experimental design and data generation process that is somewhat different from those used in previous studies [13,14]. The data generation process in [13] is not described in sufficient detail to enable a comparative analysis; however, base rates are not mentioned. In [14], 100,000 sets of four numbers (corresponding to *a*, *b*, *c*, *d*) were selected randomly from a uniform distribution subject to constraints that *a* + *b* + *c* + *d* = *n* = 1024. This process generates a very diverse set of 100,000 quadruples; however, it does not allow for an assessment of how the binary similarity coefficients compare to one another for different base rates.

We also generated 100,000 quadruples in our experiment, but did so using a rather different process. First, we selected pairs of base rates (π_1_ and π_2_) for the two attributes. Second, we generated two *n* × 1 random vectors, **x**_1_ and **x**_2_, corresponding to the base rates π_1_ and π_2_, respectively. Third, we computed *a*, *b*, *c*, and *d* corresponding to the **x**_1_ and **x**_2_ vectors. This process was repeated 100,000 times for each of 15 different pairs of base rates. The 15 base rate pairs [π_1_ and π_2_] that we used in the simulation experiment were: [.1, .1], [.1, .3], [.1, .5], [.1, .7], [.1, .9], [.3, .3], [.3, .5], [.3, .7], [.3, .9], [.5, .5], [.5, .7], [.5, .9], [.7, .7], [.7, .9], and [.9, .9]. To assure non-zero values for *a*, *b*, *c*, and *d*, we used *n* = 2000 in the data generation process.

The simulation experiment was conducted in MATLAB and the m-file used to generate the 100,000 trials for each pair of base rates is available at https://figshare.com/articles/Binary_Similarity_Coefficients_Article_-_Brusco_Cradit_Steinley/12234716. The generation of 100,000 trials for 15 different base rate pairs results in a total of 1.5 million quadruples (*a*, *b*, *c*, *d*). For each of these quadruples, we computed the 71 binary similarity coefficients. Subsequently, for each base-rate pair, we obtained the 71 × 71 correlation matrix based on the 100,000 trials for that pair. For most of the base-rate pairs, the correlations between the Goodman and Kruskal I coefficient and all other coefficients were reported as ‘NaN’ (not a number, or undefined). The same was true for the Anderberg coefficient, which is based on the Goodman and Kruskal I coefficient. The Goodman and Kruskal I and Anderberg coefficients were dropped from the study because these two coefficients were always zero for many of the base-rate pairs and, therefore, it was not possible to compute correlations between these and other coefficients. We proceeded with analysis of 69 × 69 correlation matrices throughout the remainder of the analyses.

One preliminary finding from the study was that, for each pair of base rates, there was *perfect* correlation between several subsets of binary similarity coefficients: (i) {Sokal and Michener, Hamann}, (ii) {Rogot and Goldberg, Scott}, (iii) {Gower and Legendre, Sokal and Sneath II), (iv) {Kulczynski II, McConnaughey, Johnson}, (v) {Baroni-Urbani and Buser I, Baroni-Urbani and Buser II}, and (vi) {Gleason, Van der Maarle}. These findings are consistent with those reported in [14, p. 2891).

### Partition agreement for different base-rate pairs

The next step of the analysis was to obtain, for each of the 15 base-rate pairs, a partition of the binary similarity coefficients into two clusters, based on the correlation matrix. We selected *K* = 2 clusters for two reasons. First, most of the improvement in the clustering index value for *K*-median partitioning occurs when moving from one to two clusters. Second, trying to select the ‘best’ number of clusters for each of 15 different base rate pairs adds a lot of subjectivity to the analysis and is apt to lead to a comparison that is much more confusing. The *K*-median clustering method described in the previous section was applied to the correlation matrix for each base-rate pair under the assumption of *K* = 2 clusters. The agreement between each of the 15(14)/2 = 105 pairs of two-cluster partitions was computed using the adjusted Rand index [ARI: 40]. Although the ARI is one of the binary similarity coefficients evaluated in the study, its most important role is that it is the gold standard for measuring partition agreement [41–43]. The ARI achieves a value of one for perfect agreement between two partitions and a value near zero for chance agreement. In [41], thresholds of .65, .80, and .90 for ‘fair’, ‘good’, and ‘excellent’ agreement, respectively.

Table 2 provides the ARI values between all pairs of partitions. Along the main diagonal of Table 2 are blocks of submatrices that help to identify four groups of base-rate pairs for which partition agreement between all members of the group met the threshold for ‘fair agreement’ (i.e., > .65) or better. The 4 × 4 submatrix in the top left portion of Table 2 corresponds to four conditions where *both* base-rate pairs are comparatively low (i.e., π_1_ + π_2_ ≤ 0.6) and, therefore, we refer to this group as the *low-base-rate group*. The ARI value of .9366 between the partitions for the [.1, .1] and [.1, .3] base-rate pairs meets the threshold for excellent agreement, whereas the ARI of .8233 between the partitions for [.1, .5] and [.3, .3] is good agreement. The agreement between all of the other base-rate pairs of the four-member group is fair.

**Table 2:** Two-cluster partition agreement (as measured by the ARI) among the 15 base-rate pairs.

	[.1,.1]	[.1,.3]	[.1,.5]	[.3,.3]	[.1,.7]	[.1,.9]	[.3,.9]	[.3,.5]	[.3,.7]	[.5,.5]	[.5,.7]	[.5,.9]	[.7,.7]	[.7,.9]	[.9,.9]
[.1,.1]	**1.0000**	**.9366**	**.6576**	**.7075**	.0511	.0511	.0496	-.0248	-.0248	.0464	.0464	.0829	.0834	.1220	.1016
[.1,.3]	**.9366**	**1.0000**	**.7120**	**.7641**	.0660	.0660	.0647	-.0286	-.0286	.0365	.0365	.1012	.0660	.1012	.0825
[.1,.5]	**.6576**	**.7120**	**1.0000**	**.8239**	.0495	.0495	.0486	.2073	.2073	.2876	.2876	.0492	.0053	.0492	.0135
[.3,.3]	**.7075**	**.7641**	**.8239**	**1.0000**	.0142	.0142	.0131	.1521	.1521	.2301	.2301	.0359	.0142	.0359	.0240
[.1,.7]	.0511	.0660	.0495	.0142	**1.0000**	**1.0000**	**.6777**	.0004	.0004	.0004	.0004	.4168	.2172	.3434	.2462
[.1,.9]	.0511	.0660	.0495	.0142	**1.0000**	**1.0000**	**.6777**	.0004	.0004	.0004	.0004	.4168	.2172	.3434	.2462
[.3,.9]	.0496	.0647	.0486	.0131	**.6777**	**.6777**	**1.0000**	.0089	.0089	.0089	.0089	.6777	.4168	.5839	.4560
[.3,.5]	-.0248	-.0286	.2073	.1521	.0004	.0004	.0089	**1.0000**	**1.0000**	**.7887**	**.7887**	-.0007	.0004	-.0058	.0044
[.3,.7]	-.0248	-.0286	.2073	.1521	.0004	.0004	.0089	**1.0000**	**1.0000**	**.7887**	**.7887**	-.0007	.0004	-.0058	.0044
[.5,.5]	.0464	.0365	.2876	.2301	.0004	.0004	.0089	**.7887**	**.7887**	**1.0000**	**1.0000**	.0111	.0123	-.0007	.0196
[.5,.7]	.0464	.0365	.2876	.2301	.0004	.0004	.0089	**.7887**	**.7887**	**1.0000**	**1.0000**	.0111	.0123	-.0007	.0196
[.5,.9]	.0829	.1012	.0492	.0359	.4168	.4168	.6777	-.0007	-.0007	.0111	.0111	**1.0000**	**.6777**	**.8857**	**.7272**
[.7,.7]	.0834	.0660	.0053	.0142	.2172	.2172	.4168	.0004	.0004	.0123	.0123	**.6777**	**1.0000**	**.7783**	**.9420**
[.7,.9]	.1220	.1012	.0492	.0359	.3434	.3434	.5839	-.0058	-.0058	-.0007	-.0007	**.8857**	**.7783**	**1.0000**	**.8312**
[.9,.9]	.1016	.0825	.0135	.0240	.2462	.2462	.4560	.0044	.0044	.0196	.0196	**.7272**	**.9420**	**.8312**	**1.0000**

The four blocks highlighted in bold along the main diagonal are groups of base-rate pairs for which the partition agreement among all members of the group is .65 or larger (.65 is the guideline from [41] for fair agreement).

Moving down along the main diagonal, there is a 3 × 3 submatrix in Table 2 that corresponds to three conditions whereby π_2_ –π_1_ ≥ 0.6 and, therefore, we refer to this second group of base rate pairs as the *diverse-base-rate group*. The agreement between the partitions for two of these base-rate pairs, [.1, .7] and [.1, .9] was perfect (1.0), and their agreement with the partition for the third base-rate pair, [.3, .9] was fair.

Continuing down the main diagonal, there is a 4 × 4 submatrix in Table 2 that corresponds to what we refer to as the *mid-level-base group*. The partitions for two of the base-rate pairs in this group, [.3, .5] and [.3, .7] were identical, and the partitions for the other two-base-rate pairs in the group, [.5, .5] and [.5, .7] were also identical. The ARI of .7887 between the [.3, .5]/[.3, .7] partition and the [.5, .5]/[.5, .7] partition was near the threshold for good agreement.

Finally, there is a 4 × 4 submatrix in the bottom-right corner of Table 2 that corresponds to a group consisting of some of the higher base-rate pairs. The ARI value of .9420 between the partitions for the [.7, .7] and [.9, .9] base-rate pairs meets the threshold for excellent agreement and the ARI of .8857 between the partitions for [.5, .9] and [.7, .9] approaches the threshold for excellent agreement. The ARI of .8312 between the [.7, .9] and [.9, .9] partitions also satisfies the threshold for good agreement.

Although the agreement *within* the submatrices of the four groups of base-rates is important, what is especially striking is the fact that agreement measures outside these submatrices are generally poor. There is only one ARI value outside the main diagonal blocks that meets the threshold for fair agreement: That is, the ARI of .6777 between the [.3, .9] and [.5, .9] partition. Moreover, most of the ARI values outside of the main diagonal blocks are less than 0.1. The clear implication of these results is that the concordance of binary similarity coefficients can be profoundly affected by differences in base-rate pairs. Similar base-rate pairs tend to result in similar two-cluster partitions of the binary similarity coefficients. However, for markedly different base-rate pairs, the partitions often exhibit little more than chance agreement.

### Subsets of binary similarity coefficients

Given the discordance of some partitions of the binary similarity coefficients for different base-rate pairs, it is important to establish which subsets of binary similarity coefficients are robust to changes in base-rate pairs. Table 3 presents subsets of binary similarity coefficients that were contained in the same cluster for all 15 base-rate pairs. A few coefficients that were consistent for 14 of the 15 base-rate pairs are also identified in Table 3 and are indicated with an asterisk and italic font. Subset 1 consists of 22 coefficients, which are anchored by some of the most popular association measures {phi, tetrachoric, Yule’s Q, Yule’s W, Dispersion, Cohen}. More than half of the coefficients in Subset 1 have the term *ad*–*bc* in their numerator and two other coefficients (Forbes II and Tarwid) could be rewritten to have an *ad*–*bc* term in the numerator. As noted in [11, p. 674], *ad*–*bc* is the determinant of the contingency table and its commonness is based on its relationship to a comparison of *a* to its expectation under the assumption that the two binary vectors are independent.

**Table 3:** Subsets of coefficients that fall in the same cluster for all 15 base-rate pairs.

Subset 1	Phi	Cole I	Sokal and Sneath IV	Eyraud
	Tetrachoric	Cole II	Tarantula	Michael
	Yule’s Q	Peirce I	Gilbert and Wells	CT V
	Yule’s W	Peirce II	Maxwell and Pilliner	Tarwid
	Dispersion	Forbes I	Odds Ratio	Dennis
	Cohen	Forbes II		
**Subset 2**	Jaccard	Kulczynski I	Baroni-Urbani and Buser I	Russell and Rao
	SWJaccard	Dice II	Baroni-Urbani and Buser II	Braun-Blanquet
	Gleason	CT III	Driver and Kroeber	Van der Maarle
	Fossum	Sorgenfrei	Sokal and Sneath I	
**Subset 3**	Sokal and Michener	Hamann	Sokal and Sneath II	*Faith
	Gower and Legendre	CT I	Sokal and Sneath III	*CT IV
	Rogers and Tanimoto	CT II	Austin and Colwell	
**Subset 4**	Rogot and Goldberg	Scott	Harris and Lahey	*Sokal and Sneath V
				*Goodman and Kruskal II
**Subset 5**	Kulczynski II	McConnaughey	Johnson	*Mountford
**Subset 6**	Pearson I	Pearson II	Stiles	
**Subset 7**	Dice I	Simpson		
**Ungrouped**	Loevingers H	ARI	Fager and McGowan	Peirce III
	Gower	Rand	Hawkins and Dotson	

Note–coefficients denoted by ‘*’ share cluster membership for 14 of the 15 base-rate pairs.

Subset 2 is comprised of 15 coefficients that limit the impact of negative matches. Eleven of the members of Subset 2 are co-occurrence measures that do not incorporate negative matches (i.e., neither *a* or *d* appear in their formulas). Among the most popular of these coefficients are {Jaccard, Gleason, Driver and Kroeber, Sorgenfrei, Sokal and Sneath I, Dice II}. One of the other members of Subset 2 is the Russell and Rao coefficient, which is simply the proportion of positive matches. The logarithmic analog of the Russell and Rao coefficient, CT III, is also included in Subset 2. The remaining two members of Subset 2 are the Baroni-Urbani and Buser I and II coefficients, which significantly down-weight *d* relative to a in their numerator.

Subset 3 consists of 11 coefficients, most of which are co-occurrence measures that do include negative matches in their computation. The subset is anchored by four popular coefficients that have the term (*a* + *d*) as their numerator (Sokal and Michener, Rogers and Tanimoto, Gower and Legendre, Sokal and Sneath III). Logarithmic (CT I) and transcendental (Austin and Colwell) adaptation of the simple matching measure of Sokal and Michener are also included in Subset 3. Two other measures, Hamann and Sokal and Sneath II, have slightly modified numerators of (*a* + *d*–*b*–*c*) and 2 (*a* + *d*), respectively.

The four remaining subsets are small and the coefficients in three of these subsets tend to have strong concordance with the coefficients in either Subset 1 or Subset 2. The five coefficients in Subset 4 {Rogot and Goldberg, Scott, Harris and Lahey, Sokal and Sneath V, Goodman and Kruskal II} are also commonly included in clusters with the coefficients in Subset 1. The Rogot and Goldberg, Scott, and Harris and Lahey coefficients occur in the same cluster as the coefficients in Subset 1 for 12 of the 15 base-rate pairs, and the Sokal and Sneath V and Goodman and Kruskal II coefficients occur in the same cluster as the coefficients in Subset 1 for 13 of the 15 base-rate pairs. The two coefficients in Subset 6 {Dice I, Simpson} also occur in the same cluster as the coefficients in Subset 1 for 13 of the 15 base-rate pairs. In a similar fashion, the four coefficients in Subset 5 {Kulczynski II, Johnson, McConnaughey, Mountford} are commonly included in clusters with the coefficients in Subset 2. The Kulczynski II, Johnson, and McConnaughey coefficients occur in the same cluster as the coefficients in Subset 2 for 13 of the 15 base-rate pairs. The coefficients in Subset 7 {Pearson I, Pearson II, Stiles} are chi-square-type measures. The Pearson I and Pearson II coefficients are driven by (*ad*–*bc*)^2^ in the numerator term, and the Stiles coefficient has the term (|*ad*–*bc*|–*n*/2)^2^ in its numerator. None of the coefficients in Subset 7 are strongly tied to any of the coefficients in Subsets 1, 2, or 3. The same is true for the ungrouped coefficients {ARI, Rand, Loevinger H, Peirce III, Gower, Hawkins and Dotson, Fager and McGowan} with one exception: the Peirce III coefficient occurs in the same cluster as the coefficients in Subset 2 for 13 of the 15 base-rate pairs.

Next, we turn to a more detailed analysis of the coefficients in Subsets 1, 2, and 3. The coefficients in Subset 2 are in the same cluster as the coefficients in Subset 1 for only eight of the 15 base-rate-pair partitions. There is somewhat more consistency between the coefficients in Subset 2 and Subset 3, which are in the same cluster for 11 of the 15 base-rate-pair partitions. By contrast, the coefficients in Subset 1 are in the same cluster as the Subset 3 coefficients for only four of the 15 base-rate-pair partitions. To better understand the base-rate-pair conditions where the coefficients in Subsets 1, 2, and 3 were comparable or less comparable from one another, we selected a popular exemplar from each subset. The phi coefficient, which is the Pearson correlation coefficient between two binary vectors, was selected from Subset 1. The Jaccard coefficient was selected from Subset 2. The Sokal and Michener coefficient, which is a measure of simple matching between two binary vectors, was selected from Subset 3. Table 4 provides the correlation, *r*, between all pairs of these three exemplars for each base-rate-pair combination.

**Table 4:** Correlations between selected pairs of coefficients at all 15 base-rate pairs.

Base-rate	phi.	phi	Jaccard	phi	phi
pairs	Jaccard	Sokal-Michener	Sokal-Michener	Loevinger H	ARI
[.1, .1]	.9731	.4714	.2564	.3165	.9999
[.1, .3]	.8948	.5781	.3477	.5402	.9982
[.1, .5]	.7677	.6015	.4284	.6870	-.0018
[.1, .7]	.6011	.5808	.5328	.8042	-.9982
[.1, .9]	.3492	.4683	.7428	.9030	-.9999
[.3, .3]	.9075	.8507	.5520	.5142	.9946
[.3, .5]	.8309	.9168	.6817	.6403	-.0003
[.3, .7]	.6769	.8503	.7955	.7335	-.9946
[.3, .9]	.4043	.5797	.9242	.8049	-.9982
[.5, .5]	.8179	.9998	.8179	.5765	.0055
[.5, .7]	.7154	.9173	.9077	.6425	-.0041
[.5, .9]	.4545	.6006	.9731	.6870	.0009
[.7, .7]	.6787	.8510	.9632	.5147	.9947
[.7, .9]	.4788	.5776	.9919	.5390	.9982
[.9, .9]	.4241	.4663	.9989	.3164	.9999

The phi and Jaccard coefficients have their strongest level of concordance at the lower base rates, and also tend to be stronger when the base rates are more comparable in magnitude. The largest (*r* = .9731) correlation between these two coefficients occurs for the base-rate pair [.1, .1]. The correlation (*r* = .9075) remains strong for the base-rate pair [.3, .3]. For the base-rate pair [.5, .5], correlation dips to (*r* = .8309) and, subsequently to (*r* = .6787) for [.7, .7]. The correlation for the base-rate pair [.9, .9] is poor (*r* = .4241). The propensity for the concordance between the phi and Jaccard coefficients to weaken as the base rates become more disparate is also easily observed. For example, the weakest correlation (*r* = .3492) occurs for the most disparate base-rate pair [.1, .9], and the second weakest correlation (*r* = .4043) occurs for the base-rate pair [.3, .9].

Table 4 clearly shows that the correlation between the Jaccard and Sokal and Michener coefficients becomes stronger as the base rates increase. The smallest (*r* = .2564) pairwise correlation between these two coefficients occurs for the base-rate pair [.1, .1]. The largest (*r* = .9989) pairwise correlation between the Jaccard and Sokal and Michener coefficients occurs for the base-rate pair [.9, .9]. There are six base-rate pairs where the correlation between these two coefficients is *r* ≥ .9.

The correlation between the phi and Sokal and Michener coefficients is somewhat weaker, as a correlation of 0.7 or larger is only achieved for 6 of the 15 base-rate pairs. The strongest correlation occurs when the base-rates for the two samples are close to 0.5. The correlation between the phi and Sokal and Michener coefficients is near-perfect (*r* = .9997) for the base-rate pair [.5, .5], and is also quite strong for the base-rate pairs [.3, .5] (*r* = .9168) and [.5, .7] (*r* = .9173). However, when moving farther away from base rates of .5, the correlations quickly begin to fall. The correlation values also convey the strong symmetry of agreement about the [.5, .5] base rate pair. Symmetry is evident from the fact that: (i) the correlations for base-rate pairs [.1, .1] and [.9, .9] are nearly the same, (ii) the correlations for base-rate pairs [.1, .3] and [.7, .9] are nearly the same, (iii) the correlations for base-rate pairs [.1, .5] and [.5, .9] are nearly the same, (iv) the correlations for base-rate pairs [.1, .7] and [.3, .9] are nearly the same, and (v) the correlations for base-rate pairs [.3, .3] and [.7, .7] are nearly the same.

Table 4 also provides comparisons for the phi coefficient with two popular coefficients that were in the ungrouped category in Table 2: (i) Loevinger’s H and (ii) ARI. Loevinger’s H is a widely used coefficient in Mokken scaling analysis [1]. The results in Table 4 reveal that the strongest correlation between the phi and Loevinger’s H coefficients occurs when the base rates are most disparate. The largest (*r* = .9030), second largest (*r* = .8049), and third largest (*r* = .8042) correlations between these two coefficients occurred for the [.1, .9], [.3, .9], and [.1, .7] base-rate pairs, respectively. Like the relationship between the phi and Sokal and Michener coefficients, there was also a marked symmetry between the phi and Loevinger’s H coefficients. The nature of the correlations between the phi and ARI coefficients was particularly fascinating. There is virtually no correlation (|*r*| < .01) between these two coefficients for the five base-rate pair conditions when one or both of the base rates was .5. For the six base-rate pairs where either π_1_ ≤ π_2_ ≤ 0.3 or π_2_ ≥ π_1_ ≥ 0.7, the correlation approached +1 (*r* > .99). However, for the four base-rate pairs where π_1_ ≤ 0.3 and π_2_ ≥ 0.7, the correlation approached -1 (*r* < -.99).

## Conclusions

### Summary

There are numerous applications in the psychological sciences that require the analysis of an *n* × *p* binary matrix. When the focus in on the attributes, a preliminary step is the preparation of a *p* × *p* similarity matrix. This can be accomplished using any one of several dozen available binary similarity coefficients. The coefficients can be distinguished on different characteristics, such as: (i) whether they are association or co-occurrence measures, and (ii) whether they retain or exclude information pertaining to negative matches.

Although there have been two recent surveys [13,14] that provide correlation-based groupings of binary similarity coefficients, neither study provided an assessment of how the agreement of the coefficients is affected by base rates. To address this issue, we conducted a simulation experiment that carefully controlled for base rates in the experimental design. More specifically, two-cluster *K*-median partitions of 69 binary similarity coefficients were obtained based on their inter-coefficient correlations (computed across 100,000 samples) for 15 different combinations of base-rate pairs. A succinct summary of the results of that experiment is as follows:

1. There were four groups of base-rate pairs whereby the level of partition agreement between all pairs in the group was at least ‘fair’ based on the ARI standards published by Steinely (2004): (a) {[.1, .1], [.1, .3], [.1, .5], [.3, .3]}, (b) {[.1, .7], [.1, .9], [.3, .9]}, (c) {[.3, .5], [.3, .7], [.5, .5], [.5, .7]}, and (d) {[.5, .9], [.7, .7], [.7, .9], [.9, .9]}.
2. With only one exception, the ARI between the base-rate pairs not in the same group was below the ARI threshold for ‘fair’ and, in most instances the ARI was less than 0.1, thus suggesting only chance agreement.
3. There were three sizable subsets of coefficients that were in the same cluster of the *K*-median partition for all 15 base-rate pairs. These include a subset anchored by popular association coefficients {phi, tetrachoric, Yule’s Q, Yule’s W, Dispersion, Cohen}, a subset anchored by co-occurrence coefficients that do not incorporate negative matches {Jaccard, Gleason, Driver and Kroeber, Sorgenfrei, Sokal and Sneath I, Dice II}, and a subset anchored by co-occurrence coefficients that do incorporate negative matches{Sokal and Michener, Rogers and Tanimoto, Gower and Legendre, Sokal and Sneath II, Sokal and Sneath III)}.
4. The correlations between coefficients in different subsets were quite strong for some base-rate pairs, but weak for others. Table 4 was useful for disentangling the base-rate conditions for which coefficients for the different subsets tended to be strong or weak.

The key finding of the simulation study is that base rates do matter when comparing binary similarity coefficients. The agreement between some subsets of coefficients is robust to changes in the base rates; however, the agreement between other subsets is highly sensitive to changes.

### Implications for analysis of real psychological data sets

We do not contend that base rates should be the primary factor for selecting a binary similarity coefficient for psychological applications. Instead, the context of the particular application is much more important. Nevertheless, information about the base rates does offer researchers some guidance as to when two different coefficients are likely to produce similar results. To illustrate, we consider two different psychological applications discussed earlier in the paper: (1) item-scale development and (2) psychopathology networks.

In the first application context [2,4], the data pertained to the performance of schoolchildren on 12 transitive reasoning problems. The binary measurements indicated whether a student got a problem right (*x*_*ij*_ = 1) or wrong (*x*_*ij*_ = 0). Accordingly, these data are not of the ‘attribute presence or absence’ variety, but rather reflect performance-based measurements. The base rates for the 12 problems ranged from 30.1% (hardest problem) to 97.4% (easiest problem) with an average of 74.5%. In light of the lack of presence/absence interpretation and the relative ‘easiness’ of the problems, it is arguable that the zeros in the raw data matrix should be considered at least as important as the ones. This might suggest that association coefficients or, possibly, cooccurrence coefficients that include *d* might be useful for this application.

Five-cluster partitions of the 12 transitive reasoning problems were obtained in [2,4] based on Mokken scaling analysis using the Loevinger H coefficient. A similar five-cluster partition was obtained using the *K*-median method based on the association measures tetrachoric correlation and Yule’s Q. By contrast, five-cluster partitions obtained using cooccurrence measures that include *d* (e.g., Sokal-Michener) and exclude *d* (e.g., Jaccard) spuriously placed the problems with the four highest base rates in their own individual clusters, thus exhibiting a manifestation of the size effect noted by Jackson et al. (1989) [12]. The disparity between the results obtained by association and cooccurrence methods was predicted by our findings in Table 4, which shows that phi is weakly correlated to both Jaccard and Sokal-Michener when base rates are very high. The similarity of the Jaccard and Sokal-Michener results was also predicted by the results in Table 4, which shows strong concurrence between these two coefficients when base rates are high.

In the second application context [5,44], the data pertained to 18 depression/anxiety symptoms among a set of patients. Unlike the transitive reasoning data, the depression/anxiety data, which focuses on the presence or absence of symptoms, does comport more with the ‘attribute presence/absence’ interpretation. The base rates for the depression/anxiety data ranged from 10.3% (least prevalent symptom) to 51.5% (easiest problem) with an average of 20.8%. This average is less than one-third of the corresponding figure for the transitive reasoning data. Given the presence-absence interpretation of the data and the modest prevalence of symptoms, it was argued in [44] that it is reasonable in this context to give stronger consideration to binary coefficients that ignore (or reduce the contribution of) negative matches, such as the Jaccard index. The Jaccard index (and two other coefficients from its cluster: Kulczynski II and Driver and Kroeber) led to a particularly relevant and interpretable three-cluster partition of the symptoms. Cooccurrence coefficients that include *d* (Sokal-Michener, Faith, Gower and Legendre) led to a different, yet still interpretable, partition. Thus, the size effect problem noted by Jackson et al. (1989) [12] did not manifest itself in this application context because of the lower base rates. The association coefficients (tetrachoric, Yule’s Q) led to yet a different partition that was also interpretable, but not as relevant as the one associated with the cooccurrence measures that exclude *d*. Again, the disparity among the association, cooccurrence (exclude *d*), and cooccurrence (include *d*) partitions was predicted by the results in Table 4, which shows rather lower agreement among exemplars for these three categories at low-to-moderate base rates.

To summarize, the selection of a binary similarity coefficient will largely be driven by the context of the particular application. Nevertheless, our results enable researchers to better understand the feasibility of different coefficient options based on base-rate information. For example, if a researcher perceives that negative matches are of comparable importance to positive matches, then the selection of an association coefficient from Subset 1 (e.g., phi) is appropriate. Our findings suggest that, if the researcher’s data approximate the (.5, .5) base-rate condition, then the researcher could replace an association coefficient from Subset 1 with a co-occurrence coefficient from Subset 3 (e.g., Sokal-Michener) and obtain comparable results. However, there is a greater discordance between coefficients from Subsets 1 and 3 as the base rates depart from the (.5, .5) condition.

In a study where the presence/absence of attributes is measured and the researcher perceives that negative matches are of lesser importance, then the selection of a co-occurrence coefficient from Subset 2 (e.g., Jaccard) is appropriate. Our results suggest that, under a low base-rate condition such as (.1, .1), it should be possible to replace the co-occurrence coefficient from Subset 2 with an association coefficient from Subset 1 (e.g., phi) and realize comparable results. Likewise, under a high base-rate conditions such as (.9, .9), it is possible to replace the co-occurrence coefficient from Subset 2 with a co-occurrence coefficient from Subset 3 (e.g., Sokal-Michener) that incorporates negative matches and obtain comparable results.

### Limitations and extensions

One of the primary intentions of this paper was to draw attention to the many different binary similarity coefficients that are available. As noted in the introduction of this manuscript, some of these coefficients (e.g., tetrachoric correlation) are well known, but most are not. We considered a sample of 71 coefficients from the broader literature (e.g., biology, ecology. etc.) that provided both breadth and depth with respect to the distinguishing features of association vs. co-occurrence and inclusion vs. exclusion of negative matches. However, our assembly of coefficients is not exhaustive and this could be perceived as one limitation of our paper.

Another potential limitation is the fact that we conducted our evaluation within the framework of two-cluster *K*-median partitioning. It might be interesting to investigate clusters of the binary similarity coefficients using other partitioning methods, or possibly alternative data analysis approaches such as multidimensional scaling.

A closely related limitation is the fact that we limited our comparisons to two-cluster partitions of the coefficients for each of the 15 combinations of base-rate pairs. As noted previously, this is somewhat justified by the fact that, for most base-rate pairs, the largest improvement in the *K*-median objective function tended to occur when moving from one to two clusters. Nevertheless, we recognize that two might not be the ‘best’ number of clusters for any given base-rate pair; however, it does facilitate a coherent comparison across the 15 base-rate pairs. Using ad hoc rules for choosing the number of clusters for each of the 15 different base-rate pairs would result in a comparative analysis that is both confusing and unwieldy, as well as sensitive to the rule used for choosing *K*.

A potential extension of our findings is to investigate the utility of using multiple coefficients as a means for building confidence in an experimental analysis (e.g., a cluster analysis, a multidimensional scaling study, etc.). For example, a researcher could conduct an experimental analysis using a well-known coefficient from each of three major categories of coefficients to establish a similarity matrix: (1) a correlation coefficient (e.g., phi), (2) a co-occurrence coefficient that includes negative matches (e.g., Sokal and Michener), and (3) a co-occurrence measure that excludes negative matches (e.g., Jaccard). If the results of the analyses are fairly robust across the three coefficients used to construct the similarity matrix, then considerable confidence is realized. Contrastingly, if there are salient differences among the analyses, then the researcher will need to give more careful consideration as to what type of similarity is most appropriate for the problem at hand.

## Appendix: 71 binary similarity coefficients

Some general definitions used in the presentation of the 71 binary similarity coefficients indices are *n* = *a* + *b* + *c* + *d*), τ_1_ = (max{*a*, *b*} + max{*c*, *d*} + max(*a*, *c*} + max{*b*, *d*}), τ_2_ = (max{*a* + *c*, *b* + *d*} + max{*a* + *b*, *c* + *d*}), *N* = *n*(*n*-1)/2, *B* = *ab*+*cd*, *C* = *ac* + *bd*, *D* = *ad* + *bc*, *A* = *N*–*B*–*C*–*D*, and π_1_ ≤ π_2_ are the base rates for the two binary random variables. The first 18 measures are co-occurrence coefficients that do not consider negative matches, *d* (we note that coefficients that include *n*, by definition, include *a*, *b*, *c*, and *d*). Coefficients A.19 and A.20 involve the ratio of *a* to *n*. Coefficients A.21 to A.30 have total matches (i.e., *a*+*d*) in the numerator. The next 16 coefficients (A.31 to A.46) involve some form of *ad*–*bc* in the numerator term. Coefficients A.47 to A.49 are related association coefficients that involve *ad* and/or *bc* products. Coefficients A.50 to A.51 are, respectively, the Rand index and adjusted Rand index, which are enormously popular in the clustering literature. Coefficient A.52 is Loevinger’s H, which is popular in Mokken scaling applications. The remaining 19 coefficients are assorted co-occurrence measures.

Dice I [45]                $s_{ij}^{1}=\frac{a}{\left(a+b\right)}$                (A.1)

Dice II [45]                    $s_{ij}^{2}=\frac{a}{\left(a+c\right)}$                (A.2)

Jaccard [6]                    $s_{ij}^{3}=\frac{a}{\left(a+b+c\right)}$                (A.3)

SWJaccard [6]                    $s_{ij}^{4}=\frac{3a}{\left(3a+b+c\right)}$                (A.4)

Gleason [46]                    $s_{ij}^{5}=\frac{2a}{\left(2a+b+c\right)}$                (A.5)

Kulczynski I [27]                    $s_{ij}^{6}=\frac{a}{\left(b+c\right)}$                (A.6)

Kulczynski II [27]                    $s_{ij}^{7}=\frac{1}{2}\left(\frac{a}{\left(a+b\right)}+\frac{a}{\left(a+c\right)}\right)$                (A.7)

Driver and Kroeber [47]/Ochiai [28]                    $s_{ij}^{8}=\frac{a}{\sqrt{\left(a+b)(a+c\right)}}$                (A.8)

Braun-Blanquet [48]                    $s_{ij}^{9}=\frac{a}{\max\{a+b,a+c\}}$                (A.9)

Simpson [49]                    $s_{ij}^{10}=\frac{a}{min\{a+b,a+c\}}$                (A.10)

Sorgenfrei [50]                    $s_{ij}^{11}=\frac{a^{2}}{\left(a+b)(a+c\right)}$                (A.11)

Mountford [51]                    $s_{ij}^{12}=\frac{2a}{\left(ab+ac+2bc\right)}$                (A.12)

Fager and McGowan [52]                    $s_{ij}^{13}=\frac{a}{\sqrt{\left(a+b)(a+c\right)}}-\frac{max(a+b,a+c)}{2}$                (A.13)

Sokal and Sneath I [31]                    $s_{ij}^{14}=\frac{a}{\left(a+2b+2c\right)}$                (A.14)

McConaughey [53]                    $s_{ij}^{15}=\frac{\left(a^{2}-bc\right)}{\left(a+b)(a+c\right)}$                (A.15)

Johnson [54]                    $s_{ij}^{16}=\frac{a}{\left(a+b\right)}+\frac{a}{\left(a+c\right)}$                (A.16)

Van der Maarel [55]                    $s_{ij}^{17}=\frac{\left(2a-b-c\right)}{\left(2a+b+c\right)}$                (A.17)

Consonni and Todeschini [56] (CT IV)                    $s_{ij}^{18}=\frac{\ln\left(1+a\right)}{\ln\left(1+a+b+c\right)}$                (A.18)

Russell and Rao [57]                    $s_{ij}^{19}=\frac{a}{n}$                (A.19)

Consonni and Todeschini [56] (CT III)                    $s_{ij}^{20}=\frac{(\ln\left(1+a\right)}{\ln\left(1+n\right)}$                (A.20)

Sokal and Michener [58]                    $s_{ij}^{21}=\frac{\left(a+d\right)}{n}$                (A.21)

Rogers and Tanimoto [59]                    $s_{ij}^{22}=\frac{\left(a+d\right)}{\left(n+b+c\right)}$                (A.22)

Sokal and Sneath II [31]                    $s_{ij}^{23}=\frac{2(a+d)}{\left(n+a+d\right)}$                (A.23)

Sokal and Sneath III [31]                    $s_{ij}^{24}=\frac{\left(a+d\right)}{\left(b+c\right)}$                (A.24)

Faith [29]                    $s_{ij}^{25}=\frac{\left(a+\frac{d}{2}\right)}{n}$                (A.25)

Gower and Legendre [10]                    $s_{ij}^{26}=\frac{\left(a+d\right)}{\left(a+d+\frac{\left(b+c\right)}{2}\right)}$                (A.26)

Gower (see [13])                    $s_{ij}^{27}=\frac{a+d}{\sqrt{\left(a+b)(a+c)(b+d)(c+d\right)}}$                (A.27)

Austin and Colwell [60]                    $s_{ij}^{28}=\frac{2}{\pi }\sin^{-1}\sqrt{\frac{\left(a+d\right)}{n}}$                (A.28)

Consonni and Todeschini [56] (CT I) $s_{ij}^{29}=\frac{\ln\left(1+a+d\right)}{\ln\left(1+n\right)}$                (A.29)

Hamann [61]                    $s_{ij}^{30}=\frac{\left(a+d-b-c\right)}{n}$                (A.30)

Peirce I [8]                    $s_{ij}^{31}=\frac{\left(ad-bc\right)}{\left(a+b)(c+d\right)}$                (A.31)

Peirce II [8]                    $s_{ij}^{32}=\frac{\left(ad-bc\right)}{\left(a+c)(b+d\right)}$                (A.32)

Yule’s Q [9]                    $s_{ij}^{33}=\frac{\left(ad-bc\right)}{\left(ad+bc\right)}$                (A.33)

Yule’s W [9]                    $s_{ij}^{34}=\frac{\left(\sqrt{ad}-\sqrt{bc}\right)}{\left(\sqrt{ad}+\sqrt{bc}\right)}$                (A.34)

Pearson I [62]                    $s_{ij}^{35}=\chi^{2}=\frac{n{\left(ad-bc\right)}^{2}}{\left(a+b)(a+c)(b+d)(c+d\right)}$                (A.35)

Pearson II [62]                    $s_{ij}^{36}=\sqrt{\left(\frac{\chi^{2}}{\left(n+\chi^{2}\right)}\right)}$                (A.36)

Phi [63]                    $s_{ij}^{37}=\phi =\frac{\left(ad-bc\right)}{\sqrt{\left(a+b)(a+c)(b+d)(c+d\right)}}$                (A.37)

Michael [64]                    $s_{ij}^{38}=\frac{4(ad-bc)}{{\left(a+d\right)}^{2}+{\left(b+c\right)}^{2}}$                (A.38)

Cole I [65]                    $s_{ij}^{39}=\frac{\left(ad-bc\right)}{\left(a+c)(c+d\right)}$                (A.39)

Cole II [65]                    $s_{ij}^{40}=\frac{\left(ad-bc\right)}{\left(a+b)(b+d\right)}$                (A.40)

Cohen [66]                    $s_{ij}^{41}=\frac{2(ad-bc)}{\sqrt{\left(a+b)(b+d)+(a+c)(c+d\right)}}$                (A.41)

Maxwell and Pilliner [67]                    $s_{ij}^{42}=\frac{2(ad-bc)}{\left(a+b)(c+d)+(a+c)(b+d\right)}$                (A.42)

Dennis (see [13]) $s_{ij}^{43}=\frac{\left(ad-bc\right)}{\sqrt{n(a+b)(a+c)}}$                (A.43)

Dispersion (see [13]) $s_{ij}^{44}=\frac{\left(ad-bc\right)}{n^{2}}$                (A.44)

Consonni and Todeschini [56] (CT V) $s_{ij}^{45}=\frac{(\ln(1+\mathrm{ad})-\ln\left(1+bc)\right)}{\ln\left(1+\frac{n^{2}}{4}\right)}$                (A.45)

Stiles [68] (see [13]) $s_{ij}^{46}=\log_{10}\frac{n{\left(|ad-bc|-\frac{n}{2}\right)}^{2}}{\left(a+b)(a+c)(b+d)(c+d\right)}$                (A.46)

Scott [69]                    $s_{ij}^{47}=\frac{4ad-{\left(b+c\right)}^{2}}{\left(2a+b+c)(2d+b+c\right)}$                (A.47)

Tetrachoric [7]                    $s_{ij}^{48}=\cos\left(\frac{180}{1+\sqrt{\frac{ad}{bc}}}\right)$                (A.48)

Odds ratio $s_{ij}^{49}=\frac{ad}{bc}$                (A.49)

Rand [70]                    $s_{ij}^{50}=\frac{\left(A+B\right)}{N}$                (A.50)

ARI [40]                    $s_{ij}^{51}=\frac{\left[N(A+D)-[(A+B)(A+C)+(C+D)(B+D)]\right]}{\left[N^{2}-[(A+B)(A+C)+(C+D)(B+D)]\right]}$                (A.51)

Loevinger’s H [71]                    $s_{ij}^{52}=1-\frac{b}{n\pi_{1}\pi_{2}}$                (A.52)

Sokal and Sneath IV [31]                    $s_{ij}^{53}=\frac{1}{4}\left(\frac{a}{\left(a+b\right)}+\frac{a}{\left(a+c\right)}+\frac{d}{\left(b+d\right)}+\frac{d}{\left(c+d\right)}\right)$                (A.53)

Sokal and Sneath V [31]/Ochiai [28]                    $s_{ij}^{54}=\frac{ad}{\sqrt{\left(a+b)(a+c)(b+d)(c+d\right)}}$                (A.54)

Rogot and Goldberg [72]                    $s_{ij}^{55}=\frac{a}{\left(2a+b+c\right)}+\frac{d}{\left(2d+b+c\right)}$                (A.55)

Baroni-Urbani and Buser I [30]                    $s_{ij}^{56}=\frac{\left(\sqrt{ad}+a\right)}{\left(\sqrt{ad}+a+b+c\right)}$                (A.56)

Peirce III [8]                    $s_{ij}^{57}=\frac{\left(ab+bc\right)}{\left(ab+2bc+cd\right)}$                (A.57)

Hawkins and Dotson [73]                    $s_{ij}^{58}=\frac{1}{2}(\frac{a}{\left(a+b+c\right)}+\frac{d}{\left(b+c+d\right)})$                (A.58)

Tarantula (see [13]) $s_{ij}^{59}=\frac{a(c+d)}{c(a+b)}$                (A.59)

Harris and Lahey [74]                    $s_{ij}^{60}=\frac{a(2d+b+c)}{2(a+b+c)}+\frac{d(2a+b+c)}{2(b+c+d)}$                (A.60)

Forbes I [75]                    $s_{ij}^{61}=\frac{na}{\left(a+b)(a+c\right)}$                (A.61)

Baroni-Urbani and Buser II [30]                    $s_{ij}^{62}=\frac{\left(\sqrt{ad}+a-b-c\right)}{\left(\sqrt{ad}+a+b+c\right)}$                (A.62)

Fossum (see [76]) $s_{ij}^{63}=\frac{{n(a-.5)}^{2}}{\sqrt{\left(a+b)(a+c\right)}}$                (A.63)

Forbes II [77]                    $s_{ij}^{64}=\frac{\left(na-(a+b)(a+c)\right)}{n(\min\left(a+b,a+c\right))-(a+b)(a+c)}$                (A.64)

Eyraud [78]                    $s_{ij}^{65}=\frac{n^{2}(na-(a+b)(a+c))}{\left(a+b)(a+c)(b+d)(c+d\right)}$                (A.65)

Tarwid [79]                    $s_{ij}^{66}=\frac{na-(a+b)(a+c)}{na+(a+b)(a+c)}$                (A.66)

Goodman and Kruskal I [80]                    $s_{ij}^{67}=\frac{\tau_{1}-\tau_{2}}{2n-\tau_{2}}$                (A.67)

Anderberg [81]                    $s_{ij}^{68}=\frac{\tau_{1}-\tau_{2}}{2n}$                (A.68)

Goodman and Kruskal II [80]                    $s_{ij}^{69}=\frac{\left(2\;\min\left(a,d\right)-b-c\right)}{\left(2\;\min\left(a,d\right)+b+c\right)}$                (A.69)

Gilbert and Wells [82]                    $s_{ij}^{70}=\log a-\log n-\log\left(\frac{a+b}{n}\right)-\log\left(\frac{a+c}{n}\right)$                (A.70)

Consonni and Todeschini II [56] (CT II) $s_{ij}^{71}=\frac{(\ln(1+n)-\ln\left(1+b+c)\right)}{\ln\left(1+n\right)}$                (A.71)
